# Importance of the intellectual property system in attempting compulsory licensing of pharmaceuticals: a cross-sectional analysis

**DOI:** 10.1186/s12992-019-0485-7

**Published:** 2019-06-27

**Authors:** Kyung-Bok Son

**Affiliations:** College of Pharmacy, Ewha Womans University, 52Ewhayeodae-gil, Seodaemun-gu, Seoul, 03760 South Korea

**Keywords:** Compulsory licensing, Access to medicines, Intellectual property, Patent system

## Abstract

**Background:**

Recently, interest in compulsory licensing of pharmaceuticals has been growing regardless of a country’s income- level. We aim to investigate the use of compulsory licensing as a legitimate part of the patent system and tool for the government to utilize by demonstrating that countries with a mature patent system were more likely to utilize compulsory licensing of pharmaceuticals.

**Methods:**

We used a multivariate logistic model to regress attempts to issue compulsory licensing on the characteristics of the intellectual property system, controlling for macro context variables and other explanatory variables at a country level.

**Results:**

A total 139 countries, selected from members of the World Trade Organization, were divided into a CL-attempted group (*N* = 24) and a non-CL-attempted group (*N* = 115). An attempt to issue compulsory licensing was associated with population (+) and a dummy variable for other regions, including Europe and North America (−). After controlling for macro context variables, mature intellectual property system was positively associated with attempting compulsory licensing.

**Conclusions:**

Our study provided evidence of an association between attempting compulsory licensing and matured patent systems. This finding contradicts our current understanding of compulsory licensing, such as compulsory licensing as a measure to usurp traditional patent systems and sometimes diametrically opposed to the patent system. The findings also suggest a new role of compulsory licensing in current patent systems: compulsory licensing could be a potential alternative or complement to achieve access to medicines in health systems through manufacturing and exporting patented pharmaceuticals.

## Background

Compulsory licensing, primarily attempted in low- and middle-income countries (LMICs) for HIV/AIDS treatments [1, 2], occurs when a government grants a license to regulate the enforcement of intellectual property, including patents and copyrighted works. Compulsory licensing of patented pharmaceuticals is not only deemed essential but also perceived as an available limited governmental measure that can be used to intervene in the case of market failure. Many studies have reported that the use of compulsory licensing is limited and sporadic. However, the Agreement on Trade-Related Aspects of Intellectual Property Rights (TRIPS) stated that a government can authorize the use of a patent of pharmaceuticals for its own purposes, which is commonly referred to as government use, to address public health problems [1, 3]. Therefore, member countries of the World Trade Organization (WTO) could implement the TRIPS, including government use, through national patent legislation [4]. A government-use license could be assigned either to a government entity or non-government entities. Furthermore, compulsory licensing could occur following the request by several stakeholders after failure to obtain a voluntary license. These various forms of compulsory licensing could be utilized for the domestic market and foreign market, including exporting pharmaceuticals to low- and middle-income countries (LMICs) [1, 3]. Notably, adequate remuneration is required in these cases [3, 5].

Recently, interest in the compulsory licensing of pharmaceuticals has been growing regardless of a country’s income level. The UN High-Level Panel on Access to Medicines recommended the implementation of legislation and use of compulsory licensing, which is one of the notable flexibilities of TRIPS, for legitimate public health needs [6]. Furthermore, the Lancet Commission on Essential Medicines Policies recommended that national patent legislation allow for effective licensing of essential medicines in the absence of voluntary licensing [7]. Finally, the European Parliament, notably including several high-income countries, adopted a resolution on options for issuing compulsory licensing for European Union Member States [8]. At the same time, the pledge not to use compulsory licensing to lower domestic drug prices was taken by many high-income countries after the Declaration on the TRIPS Agreement and Public Health (the Doha Declaration) [9, 10].

Although compulsory licensing is recognized as a legal measure against patent abuse and public health problems, there has been continuous debate regarding compulsory licensing between high-income countries and LMICs. The basic argument in favour of compulsory licensing stems from the availability of affordable and essential medicines for improved public health [11]. Public health and greater humanitarian approaches to access to medicines were the driving forces behind the Doha Declaration that clearly reaffirmed compulsory licensing as a right of member countries of the WTO [12]. Additionaly, some argue that compulsory licensing is needed to counter high-income countries’ obstinacy for intellectual property rights as new norms of international trade agreements [11]. Furthermore, they argue that too many intellectual property rights could result in less innovation [13].

On the other hand, others argued that the rights behind patents are historically and statutorily granted with the issuance of a patent and should be protected through a limited time period [11, 14]. For instance, the patent system in the United States grants the right of the patent holder to exclude others from making, using, offering for sale, or selling the intervention throughout the United States or importing the invention into the United States. Furthermore, some insist that compulsory licensing does not allow the patent holder to recoup any investment incurred through research and development and to make a sufficient profit for them to remain in business [14]. They argue that patents are granted to inventors to exclude others to provide these incentives. Therefore, opponents deem compulsory licensing as a measure to usurp traditional patent systems, sometimes diametrically opposed to the patent system, and diminishing the incentives for innovative medicines for all humanity.

These normative arguments provide clues to partially understand the validity of compulsory licensing and reason for its existence in the patent system. In line with these debates, evidence, specifically from empirical literature, is still needed to understand the association between compulsory licensing and the intellectual property system, particularly the patent system. However, few literature sources are available, even non-empirical literature that provide associations between compulsory licensing and the patent system at the country level. For instance, a recent study summarized notable factors significantly influencing the issuance of compulsory licensing based on previous literature: local manufacturing capacity or importing possibilities to supply medicines and pressure from patent holders’ threat of market withdrawal [15]. Additionally, Ford et al. (2007) suggested three factors sustaining access to antiretroviral therapy in Brazil and Thailand: legislation for access to medicines, public sector capacity to manufacture medicines, and strong civil societies to support government [16]. Paradoxically, the patent system itself has not been treated importantly to understand the compulsory licensing of patents.

However, we should note that the compulsory licensing of patents was originally discussed and legislated in high-income countries, including the United States, Germany, and England with the development of the patent system [17, 18]. For instance, the state of South Carolina in the United States enacted the Act for the Encouragement of Arts and Science in 1784. The Act provided exclusive privileges of the machine (patents) to be subject to the same privileges and restrictions imposed on the author (copyrights) [17]. Additionally, it could be argued that compulsory licensing positively influenced the survival of certain patents or patent systems because compulsory licensing was granted as an alternative to the abolition of patents. In other words, compulsory licensing could be initiated in countries where suitable patent systems were established. However, there is a lack of empirical literature, even case studies, presenting associations between compulsory licensing and the patent system.

Given this, we aimed to empirically investigate the associations between attempts to issue compulsory licensing of pharmaceuticals and the intellectual property system and to demonstrate the positive role and function of the matured intellectual property system in attempting compulsory licensing. Not surprisingly, a close relationship exists between the patent system and compulsory licensing of patents. Issuing compulsory licensing of patents requires a granted patent, and the grant of the patent requires a functioning patent system. In this study, we investigated the use of compulsory licensing as a legitimate part of the patent system and tool for the government to utilize by demonstrating that countries with a mature patent system were more likely to utilize compulsory licensing of pharmaceuticals.

## Methods

### Variables and measurement

Given the availability of the data, we selected 139 member countries of the WTO, including observers. We examined the presence of attempted compulsory licensing of pharmaceuticals between 1995 and 2014. Attempted compulsory licensing data were obtained from a previous study [1], in which attempts based on various sources were collected from the following: documents on the webpage of civil society entities; peer-reviewed articles and grey literature; and news sources such as the Access World News Collection. Additionally, we retrieved data from the publicly available TRIPS Flexibilities database, which is available at the website of Medicines Law & Policy, to supplement our data source [19]. The TRIPS Flexibilities database notably contains various instances, including a government announcement of the intent to initiate compulsory licensing and a request or application by a third party to invoke compulsory licensing. The database was constructed by several primary sources, including non-public documents [3]. However, we excluded cases based on non-public documents in this study. After we thoroughly reviewed the instances, we included one additional case from Kenya that was confirmed in other sources.

Based on already known literature and theories [1, 2, 15, 16, 20–24], we chose a set of explanatory variables, specifically geographical, economic, and political preconditions of a country to control for their effects (Table 1). In summary, we chose variables such as region, income, population, and polity in our model as macro context variables.

**Table 1:** Description of variables and sources used in the study

Variable	Description	Sources
CL attempted	Binary variable for presence of attempted compulsory licensing occurring between 1995 and 2014	Son and Lee (2017), ‘t Hoen et al. (2018)
Region	Dummy variable for location of the country (Africa, Asia, Latin America, Others)	World Bank
Population	Natural logarithm of the number of the population in 2014	World Bank
Income	Natural logarithm of GNI per capita in 2014	World Bank
Polity	Dummy variable for annual polity scores in 2014 (Autocracy, Anocracy, Democracy)	Polity IV country report
The PIPP index	Annual index summarizing the presence, term, and strength of various types of patents that can be claimed for pharmaceutical innovations.	Liu and Croix (2014)

First, compulsory licensing has been mostly attempted in Africa, Asia, and Latin America [1, 2]. Thus, we selected a region as the dummy variable for the country location. Because only one European country was included in the database, we combined North America and Europe into the group termed ‘others’. It was also reported that the approach to compulsory licensing might vary with the country income level [1]. For instance, compulsory licensing in high-income countries might be used as a price negotiation tool. On the other hand, compulsory licensing in low-income countries might not be merely a negotiation tool but also an actual instrument to guarantee access to medicines. In line with this speculation, we included the income variable in the model.

Second, the number of patients is an important context by which limited access to medicines is perceived as a public health problem, and might be connected to attempting compulsory licensing [25]. In line with this assumption, we added the total population variable, which approximates the number of patients, in the model. Furthermore, patent holders might employ various strategies against issuing compulsory licensing to retain their profit margins in different markets [25]. For instance, patent holders might cooperate with the government and discount the price or agree to voluntary licensing to leave the threat to their potential profit unrealized in big markets comprising many patients with purchasing power [20], while patent holders forgo the profits derived from a small market with a few patients without purchasing power but still have an incentive to prevent compulsory licensing, possibly inducing other cases in other markets [25].

Third, the political system might matter in attempting compulsory licensing. For instance, the political system might induce the increased supply of public goods and public policy [26]. Additionally, politicians might attempt compulsory licensing to legitimize their political party or regime [25]. Given this, we added polity as a dummy variable in the model. The polity data series (series) measures a state’s level of democracy. Specifically, the series evaluates competitiveness, openness, and participation in a state’s political system, including civil participation [27]. Based on these criteria, a “polity score”, ranging from − 10 to 10, was determined for each year and country. In the series, there were three types of political systems: democracy with a 6 to 10 polity score; anocracy with a − 5 to 5 score; and autocracy with a − 10 to − 6 score. Specifically, anocracy means a regime with mixed democratic and autocratic features. In this study, we used the last version, Polity IV, to measure policy scores in 2014.

Finally, the Pharmaceutical Intellectual Property Protection (PIPP) index was used to identify associations between attempted compulsory licensing and the characteristics of intellectual property systems. The PIPP index summarizes the presence, term, and strength of various types of patents that can be claimed for pharmaceutical innovations [28]. Specifically, the PIPP index comprises three components: the Pharmaceutical Patent Rent Appropriation (PPRA) index; the Pharmaceutical Patent International Agreements (PPIA) index; and the Pharmaceutical Patent Enforcement (PPE) Index. The PPRA index measures the presence of various types of pharmaceutical patents that provide protection for various pharmaceutical inventions. The PPIA index presents country membership in international agreements. The PPE index measures various statutory measures that either enhance or detract from public and private enforcement of patents [28]. The PPIP index has been used in various econometric literature sources regarding corporate strategies, foreign direct investment and trade [29, 30].

### Model specification and statistical analysis

Descriptive analyses were used to present the difference between two groups, including the CL-attempted group and non-CL-attempted group. Specifically, *chi-squared* test or *Fisher’s exact* test was applied for the categorical variates and *t* test was conducted for the continuous variates to examine whether the variables of interest differed by the groups. Next, two multivariate logistic regressions were conducted to elucidate factors that affected the attempting compulsory licensing. Variables of interest such as region, population, income, and polity were added in the model to adjust for the macro context. Population and income were used after log transformation to normalize the distribution. The PIPP index was then added to answer our research questions. Furthermore, we conducted additional analysis assigning weights to the variable of income level to reflect the real world situation. Data management and analysis were performed using R statistical software (version 3.4.1). Significance was considered for *p*-values less than 0.05.

Model:

$$ \mathrm{CL}\ {\mathrm{attempted}}_{\mathrm{i}}=\kern0.5em {\upbeta}_0+{\upbeta}_1\kern0.5em {\mathrm{region}}_{\mathrm{i}}+\kern0.5em {\upbeta}_2\log \left(\mathrm{population}\right)\kern0.5em +\kern0.5em {\upbeta}_3\log \left({\mathrm{i}\mathrm{ncome}}_{\mathrm{i}}\right)+\kern0.5em {\upbeta}_4{\mathrm{polity}}_{\mathrm{i}}+\kern0.5em {\upbeta}_5{\mathrm{PIPP}}_{\mathrm{i}}+{\upvarepsilon}_{\mathrm{i}} $$

## Results

Among 139 countries, 24 attempted compulsory licensing, while 115 did not. In the CL-attempted group, the number of attempted compulsory licensing ranged from 1 to 16. The mean, median, and mode of attempted compulsory licensing were 3.9, 3 and 1, respectively.

Table 2 presents the descriptive statistics of the dependent and explanatory variables in the analysis. Significant differences were found in the region, population, and the PIPP index between the groups. First, significant difference was found in the distribution of countries in the region variable. In the CL-attempted group, 8, 7, 6, and 3 countries belonged to Africa, Asia, Latin America, and others, respectively, while 45, 16, 15, and 39 countries belonged to the same category in the non-CL-attempted group. Second, a significant difference was noted in the population between the groups. The mean of the log-transformed population was 17.82 in the CL-attempted group and 16.24 in the non-CL-attempted group. Finally, the PIPP index, which is our main interest of the study, was significantly different. The PIPP index was 2.13 and 1.61 for the CL-attempted group and non-CL-attempted group, respectively. However, no difference was found in the other variables such as income and polity.

**Table 2:** Descriptive statistics of variables used in the study

Variable	CL-attempted group N = 24	Non CL-attempted group *N* = 115	*P* value
CL attempted	4.16 ± 4.22	0	N/A
Region			0.04312
Africa	8	45	
Asia	7	16	
Latin America	6	15	
Others	3	39	
Population (log)	17.82 ± 1.35	16.24 ± 1.28	*P* < 0.0001
Income (log)	8.58 ± 1.31	8.54 ± 1.57	0.9235
Polity			0.7944
Democracy	16	66	
Anocracy	6	38	
Autocracy	2	11	
The PIPP index	2.13 ± 0.73	1.61 ± 1.00	0.0188
range	1.13–4.51	0.00–3.45	

Table 3 presents multivariate logistic regression for attempted compulsory licensing in 139 countries. Note that we have two models with two different databases for the analysis. Specifically, model 1 does not include the weight to the variable of income, while model 2 applied weights to the same variable. We used the database constructed by Son and Lee (2018) to detect the presence of attempted compulsory licensing of pharmaceuticals between 1995 and 2014. To complement the database, we used the TRIPS flexibilities database, which is publicly accessible at the website of Medicines Law and Policy.

**Table 3:** Factors affecting the attempt to issue compulsory licensing

Model	Model 1: Without weights to the variable of income						Model 2: With weights to the variable of income					
Data source	Son and Lee (2018)			Updated information with ‘t Hoen et al. (2019)			Son and Lee (2018)			Updated information with ‘t Hoen et al. (2019)		
index	Estimate	SE	P value	Estimate	SE	P value	Estimate	SE	P value	Estimate	SE	P value
Region *(Ref. Others)*												
Africa	2.4587	1.1664	0.0350	2.7947	1.1915	0.0189	2.1885	0.3819	< 0.0001	2.4772	0.3875	< 0.0001
Asia	1.9965	1.0580	0.0591	2.0194	1.0789	0.0612	1.7490	0.3345	< 0.0001	1.7563	0.3398	< 0.0001
Latin America	3.1951	1.1070	0.0038	3.2869	1.1325	0.0037	3.0282	0.3583	< 0.0001	3.0871	0.3647	< 0.0001
Population (log)	0.9215	0.2580	0.0003	0.9764	0.2672	0.0002	0.9565	0.0889	< 0.0001	0.9994	0.0914	< 0.0001
Income (log)	0.1105	0.2662	0.6781	0.0600	0.2647	0.8205	0.0367	0.0930	0.6930	−0.0191	0.0923	0.8356
Polity *(Ref. Anocracy)*												
Autocracy	0.3731	1.3486	0.7820	0.5930	1.3779	0.6669	0.2539	0.4749	0.5930	0.4560	0.4845	0.3466
Democracy	0.2591	0.7457	0.7281	0.5884	0.7391	0.4259	0.3651	0.2693	0.1750	0.6697	0.2684	0.0126
The PIPP index	0.8511	0.4036	0.0349	0.9315	0.4109	0.0234	0.7793	0.1393	< 0.0001	0.8505	0.1411	< 0.0001

In model 1 with two different data sources, we found consistent results. Specifically, the regions, including Africa and Latin America (reference others), populations, and the PIPP index were significant factors in attempting compulsory licensing. However, the variables of income and polity were not significant factors. Specifically, there was a higher likelihood of attempting compulsory licensing in African and Latin American countries compared than in other regions such as Europe and North America. The population was positively associated with attempting compulsory licensing. Interestingly, we found that the PIPP index was positively associated with attempting compulsory licensing. In other words, countries with mature patent systems had a higher likelihood of attempting compulsory licensing in our model. In model 2, in which weights were applied to the variable of income in the model, we found the similar results. The significant variables in model 1, such as the region, population and the PIPP index, were also significant variables in model 2. Additionally, a higher likelihood exists for attempting compulsory licensing in Asian countries than that in other regions such as Europe and North America.

## Discussion

Compulsory licensing could be issued by a government in situations where the patent holder is either not using the patent or is not using it adequately, including pharmaceuticals in demand that are not fully available. When governments attempt to issue compulsory licensing or non-governmental entities request a government to issue compulsory licensing, the outcome is often a decrease in prices, similar to the case of the introduction of generics in the market [16, 31]. Although compulsory licensing is not a new concept, it has received considerable debates as patent holders seek to advance their political stances over intellectual property rights to access to medicines. Specifically, opponents of compulsory licensing argue that compulsory licensing would be diametrically opposed to the function of the patent system.

We designed this study to demonstrate the positive role of the matured intellectual property system in attempting compulsory licensing. The issuance of the compulsory licensing of a patent requires a granted patent, which is the outcome of a functioning patent system. Therefore, a link exists between the compulsory licensing of patents and the patent system. In this study, we presented that the use of compulsory licensing is an essential part of the patent system and a legitimate tool for the government to utilize by demonstrating that compulsory licensing of pharmaceuticals is utilized in particular countries with a mature patent system.

To our knowledge, this study is the first to empirically analyse the relationship between compulsory licensing and patent systems. We used a multivariate logistic model to regress the attempt to issue compulsory licensing on the characteristics of the intellectual property system (i.e., the PIPP index), controlling for macro context variables (i.e., region, population, income, and polity) at a country level. Interestingly, mature patent systems were positively associated with attempting compulsory licensing. How then might mature patent systems affect the issuance of compulsory licensing?

The history of compulsory licensing might help to explain these interesting results because they provide a reason for the existence of compulsory licensing in a patent system. We will trace the origins of the compulsory licensing of patents in high-income countries and discuss interesting cases from Canada in the late 1980s.

### History of compulsory licensing of patents

The following legislative cases in the 18-nineteenth century demonstrate that high-income countries actively reviewed and introduced compulsory licensing to complement the intellectual property system and develop their own industrial policy.

The compulsory licensing of patents was followed by the compulsory licensing of copyrights [11]. In England, excessive price and an insufficient supply by monopolies were recognized as an economic disease [32]. The Statute of Anne in 1710, known as the world’s first copyright law, reflected this thought. Someone other than the owner of the copyright who determined that a book was over-priced could file a complaint against the owner of the copyright with the court, and a fine was imposed to the owner of the copyright if it violated the price set by the court. Following the Statute of Anne, the United States, specifically the state of Connecticut, established the Copyright Act in 1783. The Copyright Act required copyrighted books to be sold at a reasonable price in sufficient quantities; otherwise, it would be possible to file a complaint with the court, similar to the situation of the Statute of Anne. Furthermore, the court had the authority to determine the quantity and price for the copyright owner; if the copyright owner did not accept or rejected such a request, the court had the authority to have the complainant print it. Therefore, the Copyright Act was evaluated as a legislative example of the world’s first system of compulsory licensing of copyrights [17].

The United States, specifically the state of South Carolina, established the Act for the Encouragement of Arts and Science, providing privileges and restrictions imposed to authors (copyrights) as well as to inventors (patents) in 1784. The Act first devised compulsory licensing as a new remedy against the abuse of the patents when only invalidation of the patents existed. It should also be noted that the United States had been more aggressive in dealing with patent abuse during the development of new industry. Similarly, England and Germany discussed compulsory licensing as measures to minimize the adverse effects of the patent system and complement the shortcomings of the patent system in 1851 and 1853, respectively [18].

These trends favouring compulsory licensing in various fields persisted in the 1900s [5, 33]. The United States invoked government use on a regular basis to import pharmaceuticals in the 1960s. Furthermore, Canada utilized the compulsory licensing of pharmaceuticals not only for importation but also for local production to manage health expenditure [5, 34, 35]. For instance, the Canadian government enacted compulsory licensing by amending the Patent Act to encourage local manufacturing of the pharmaceutical and market competition in 1923 [5]. Furthermore, there was public concern on the price of pharmaceuticals that was deemed as excessive by the 1960s. Given the concern, the government made several amendments to the Patent Act, which would have significant implications on patenting activities. Additionally, the amendments allowed the use of compulsory licensing to manufacture or import pharmaceuticals that were restricted to manufacturing before amendments [5]. The benefits of compulsory licensing are palpable in Canada, and a number of follow-on drugs have entered the market.

However, the positive stance regarding compulsory licensing of high-income countries notably changed in the twentieth century, specifically during the negotiations for TRIPS [36]. LMICs argued for the right to issue compulsory licensing for patented pharmaceuticals that were expensive for their citizens, while high-income countries, usually influenced by the pharmaceutical industry, argued for the limited use of compulsory licensing [37]. High-income countries feared that massive issuance of compulsory licensing would adversely impact the profits of the industry and would harm the ability of these companies to research and develop in the long term [36, 38]. Therefore, the use of compulsory licensing of pharmaceuticals was restricted to highly infectious diseases such as HIV/AIDS in LMICs [39], and compulsory licensing other than this restricted area was deemed unjustifiable [38].

Meanwhile, WHO adopted the Doha Declaration at the fourth Ministerial Conference, and affirmed that compulsory licensing is the right of the member countries of the WTO. The Doha declaration confirmed that compulsory licensing is intended as an efficient and straightforward instrument for LMICs to improve access to needed medicines [34]. The Doha declaration was quite effective in issuance of compulsory licensing in the short run [1]. A few attempts to issue compulsory licensing, including cases from low-income countries, occurred after the declaration. However, the benefits of the declaration in issuing compulsory licensing was not sustained in the long term [1].

### Role of compulsory licensing in patent systems and health systems

Our findings suggested a new role of compulsory licensing in current patent systems. Compulsory licensing is alternatively or complementally used in countries where mature patent systems were established. For instance, China released “Measures for Compulsory Licensing of Patent Implementation” that integrated and updated intellectual property laws to allow compulsory licensing in 2012 [40]. Under the new measures, China’s State Intellectual Property Office (SIPO) may issue and terminate compulsory licensing for patents [41].

Furthermore, compulsory licensing in the patent system could be harmonized with the health system to secure access to essential medicines. In 2016, for the first time, the German Federal Patent Court notably ordered a provisional compulsory license under Section 24 of the Patent Act. The Court allowed a license seeker to continue to market an HIV drug that was made and sold since 2008 in Germany. Prior to that, the patent holder had requested a preliminary injunction against a license seeker in 2015 based on an active patent granted in 2011, partially covering the drug. The license seeker offered a voluntary license on the patent to the patent holder, but failed. The license seeker then responded by requesting a compulsory license under the Patent Act [42]. Specifically, the Court concluded that the license seeker was entitled to emergency relief and granted provisional compulsory licensing based on the need of patients to have the drug available continuously during the course of treatment [38].

Finally, compulsory licensing could be utilized to export pharmaceuticals to other countries according to paragraph 6 of the Doha declaration. However, cases on the compulsory licensing of pharmaceuticals for export are rare [33]. Meanwhile, an amendment to the TRIPS was enacted on January 23, 2017. This amendment allowed countries to grant compulsory licensing to generic suppliers exclusively to manufacture and export medicines to countries lacking production capacity [43]. It provides a secure legal basis for both potential importers and exporters to adopt domestic legislation, and establishes the means to allow countries to import affordable follow-on drugs from countries where pharmaceuticals are patented.

### Limitations of the study

This study possesses several limitations. First, this study used the presence of the attempted compulsory licensing of pharmaceuticals between 1995 and 2014. The number of countries that attempted compulsory licensing during the study period was 24, comprising approximately 20% of the total of 139 countries analysed. Therefore, some may argue that compulsory licensing is not suitable for quantitative research. However, it should be noted that empirical research might provide more practical implications on issuing compulsory licensing in the real world. This research will contribute to expanding the current understanding of compulsory licensing. Second, because of scant available data and fewer cases on compulsory licensing, a cross-sectional analysis was applied to investigate the associations between attempting compulsory licensing and intellectual property systems. However, panel analysis might be a more accurate method to understand these associations. Third, this study used the database constructed by Son and Lee (2018) to detect the presence of the attempted compulsory licensing of pharmaceuticals occurring between 1995 and 2014 [1]. Meanwhile, a more comprehensive database regarding TRIPS flexibilities, including compulsory licensing, was publicly available [19]. We supplemented the database for the analysis. However, we excluded cases from Mongolia, Pakistan, Papua New Guinea, and the Philippines that were based on non-publicly available documents such as patent letters held by procurement agencies.

## Conclusions

Our study provided evidence of an association between attempting compulsory licensing and matured patent systems. Specifically, we demonstrated that the use of compulsory licensing is an essential part of the patent system and a legitimate tool for the government to utilize. This finding contradicts our current understanding of compulsory licensing, such as compulsory licensing as a measure to usurp traditional patent systems and sometimes diametrically opposed to the patent system. Furthermore, the findings suggest a new role of compulsory licensing in current patent systems: compulsory licensing could be a potential alternative or complement to achieve access to medicines in health systems through manufacturing and exporting patented pharmaceuticals.

## Data Availability

Not applicable.
